# Transcriptomic analysis of *Rhizobium leguminosarum* bacteroids in determinate and indeterminate nodules

**DOI:** 10.1099/mgen.0.000254

**Published:** 2019-02-19

**Authors:** R. T. Green, A. K. East, R. Karunakaran, J. A. Downie, P. S. Poole

**Affiliations:** ^1^​ John Innes Centre, Norwich Research Park, Norwich NR4 7UH, UK; ^2^​ Department of Plant Sciences, University of Oxford, South Parks Road, Oxford OX1 3RB, UK

**Keywords:** *Rhizobium leguminosarum*, determinate nodules, indeterminate nodules, bacteroids, transcriptomics, RNA- Seq

## Abstract

Two common classes of nitrogen-fixing legume root nodules are those that have determinate or indeterminate meristems, as in *Phaseolus* bean and pea, respectively. In indeterminate nodules, rhizobia terminally differentiate into bacteroids with endoreduplicated genomes, whereas bacteroids from determinate nodules are less differentiated and can regrow. We used RNA sequencing to compare bacteroid gene expression in determinate and indeterminate nodules using two *Rhizobium leguminosarum* strains whose genomes differ due to replacement of the symbiosis (Sym) plasmid pRP2 (strain Rlp4292) with pRL1 (strain RlvA34), thereby switching symbiosis hosts from *Phaseolus* bean (determinate nodules) to pea (indeterminate nodules). Both bacteroid types have gene expression patterns typical of a stringent response, a stressful environment and catabolism of dicarboxylates, formate, amino acids and quaternary amines. Gene expression patterns were indicative that bean bacteroids were more limited for phosphate, sulphate and iron than pea bacteroids. Bean bacteroids had higher levels of expression of genes whose products are predicted to be associated with metabolite detoxification or export. Pea bacteroids had increased expression of genes associated with DNA replication, membrane synthesis and the TCA (tricarboxylic acid) cycle. Analysis of bacteroid-specific transporter genes was indicative of distinct differences in sugars and other compounds in the two nodule environments. Cell division genes were down-regulated in pea but not bean bacteroids, while DNA synthesis was increased in pea bacteroids. This is consistent with endoreduplication of pea bacteroids and their failure to regrow once nodules senesce.

## Data Summary

1. The genome sequence of *Rhizobium leguminosarum* bv. *phaseoli* 4292 (Rlp4292) is available from the JGI (Joint Genome Institute); taxon ID 2516653085 (GenBank accession no. AQZR00000000).

2. The genome sequence of *R. leguminosarum* bv. *viciae* 248 is available from the JGI; taxon ID 2515075009 (GenBank accession no. ARRT00000000).

3. Updated fasta, gff and Geneious files for the genomes of Rlp4292 and RlvA34 are available from www.dropbox.com/sh/yusb7ptvzd4odun/AAA7hj5rLoGbqf30ZY0Gv7Q2a?dl=0.

4. Raw data of read counts per gene locus for each replicate for Rlp4292 and RlvA34 are provided in Table S1, available with the online version of this article).

5. Details of samples and accession codes for RNA sequencing data submitted to the European Bioinformatics Institute (EBI) database (www.ebi.ac.uk/) (study accession code PRJEB28599) are given in Table S2.

**Impact Statement**

In N_2_-fixing symbioses between legumes and rhizobia, two different nodule types are those with determinate and indeterminate nodules as seen with bean and pea, respectively. Comparative transcriptomics was carried out using two rhizobial strains sharing a common core genome but each with a different symbiosis (Sym) plasmid conferring the ability to form efficient nitrogen-fixing nodules on either bean or pea. Bacteroids in both nodule types expressed genes related to nitrogen fixation, utilization of dicarboxylates, formate and amino acids, and induction of a stringent response. However, it was evident from the patterns of gene expression that the environments within the two nodule types were different, with respect to nutrient (P, S, Fe, Mo) limitation and utilization of carbon substrates. Bean bacteroids appeared to induce many genes predicted to be associated with detoxification. Pea bacteroids had increased expression of genes associated with central metabolism, the TCA (tricarboxylic acid) cycle and bacteroid differentiation. This study provides a window into the different environments experienced by nitrogen-fixing bacteroids in these two nodule types.

## Introduction

Rhizobia are a group of α- and β-proteobacteria forming symbiotic nitrogen-fixing nodules on legumes [1]. Legume nodulation is typically initiated by exchange of signalling compounds, with rhizobia attaching to root hairs and growing down plant-made infection threads into the root cortex [2]. Rhizobia are then endocytosed and surrounded by a plant-derived membrane (symbiosome membrane). The resulting structure, which resembles an organelle, is called a symbiosome [3], within which bacteria differentiate into bacteroids. N_2_ reduced to ammonia is supplied from bacteroids to the plant in exchange for a carbon supply, mostly in the form of dicarboxylic acids, such as malate [3]. Bacteroids exist in a microoxic environment, essential for activity of the oxygen-sensitive nitrogenase [4, 5].

Nodules on phaseoloid legumes (including *Phaseolus vulgaris*) are determinate with a transient meristem; the rhizobia do not terminally differentiate and nitrogen-fixing bacteroids can be cultured from mature nodules. In contrast, nodules formed on indeterminate nodules such as pea (*Pisum sativum*) maintain an active meristem and infection zone. In indeterminate nodules, growing infection threads release rhizobia into nodule cells; these bacteria endoreduplicate their genomes and terminally differentiate into pleiomorphic nitrogen-fixing bacteroids that cannot be cultured [6]. The main body of indeterminate nodules contains nitrogen-fixing symbiosomes [2, 7]. In legumes such as *Medicago truncatula* and *Pisum sativum,* belonging to the inverted repeat-lacking clade (IRLC), there are up to 600 genes which encode nodule-specific cysteine-rich (NCR) peptides [8–10] that induce bacteroid differentiation [11, 12].

Studies on bacteroid gene expression using transcriptomic techniques such as microarrays or RNA sequencing (RNA-Seq) have compared free-living cells with bacteroids from *Rhizobium leguminosarum* bv. *viciae* 3841 [13], *Bradyrhizobium japonicum* [14–16], *Azorhizobium caulinodans* ORS571 [17], *Sinorhizobium* NGR234 [18] and *Sinorhizobium meliloti* 1021 [19–21] (for a comprehensive review see [22]). Advances in RNA-Seq have allowed analysis of the transcriptomes of both plant and bacteroids in different zones of *S. meliloti*-infected *Medicago truncatula* nodules [23].

Our aim was to compare gene expression in determinate bean and indeterminate pea bacteroids. We used two *R. leguminosarum* strains that efficiently nodulate and fix nitrogen in bean or pea nodules. They differ due to the replacement of the symbiosis (Sym) plasmid pRP2 (in strain Rlp4292) with pRL1 (in strain RlvA34) but share a core genome, facilitating direct comparison of transcriptomes in bacteroids from determinate and indeterminate nodules.

## Methods

### Bacterial strains, plasmids and bacterial cultures

*R. leguminosarum* bv. *phaseoli* strain 8002 is the parent of both strains used. Strain 4292 (Rlp4292) is a rifampicin-resistant derivative of 8002. Strain 8400 was derived from 8002 through loss of the Sym plasmid pRP2 [24]. Strain 8401 is a streptomycin-resistant derivative of 8400 [24] and *R. leguminosarum* bv. *viciae* strain A34 (RlvA34) was made by conjugating into 8401 the Sym plasmid pRL1 from *R. leguminosarum* bv. *viciae* 248 [25]. The antibiotics used were rifampicin (10 µg ml^−1^) and streptomycin (500 µg ml^−1^). *Rhizobia* were grown at 28 °C on tryptone yeast extract (TY) [26] or acid minimal salts (AMS) medium or agar [27] containing 20 mM succinate and 10 mM ammonium chloride. For RNA isolation, three cultures of Rlp4292 and RlvA34 were grown in AMS to OD_600_0.4–0.6 and 12 ml culture was added directly to 24 ml RNAlater stabilization solution (Ambion).

### Plant growth, nodule collection and bacteroid isolation

*Phaseolus vulgaris* cv. Tendergreen (dwarf bean) and *Pisum sativum* cv. Avola (pea) seeds were surface sterilized, inoculated with Rlp4292 (bean) or RlvA34 (pea) and sown in 5 l pots containing sterile 1 : 1 terra-green/sand with sterile nitrogen-free nutrient solution (1 mM CaCl_2_.2H_2_0, 100 µM KCl, 800 µM MgSO_4_.7H_2_O, 10 µM Fe EDTA, 35 µM H_3_BO_3_, 9 µM MnCl_2_.4H_2_O, 0.8 µM ZnCl_2_, 0.5 µM Na_2_MoO_4_.2H_2_O, 0.3 µM CuSO_4_.5H_2_O, 25 g KH_2_PO_4_ l^−1^ and 28.4 g Na_2_HPO_4_l^−1^). Plants were grown at 22 °C with a day–night cycle of 16 h light/8 h dark until the appearance of flowers, which occurred 35 (bean) and 28 (pea) days after inoculation. On the appearance of flowers, which corresponds to the peak of nitrogen fixation, three samples of nodules were harvested from plants (six plants per sample). Nodules were macerated with 5 ml isolation buffer (1 M K_2_HPO_4_, 1 M KH_2_PO_4_, 300 mM sucrose, 2 mM MgCl_2_) then filtered through five layers of muslin. Plant material and mature bacteroids were separated using differential centrifugation [28] and bacteroids re-suspended in 1 ml 10 mM Tris-HCl pH 8.0.

### RNA isolation, sequencing, mapping and bioinformatic analyses

Bacteroids and free-living cells were ribolysed (MP Biomedicals FastPrep) with 300 mg 0.1 mm silica and 100 mg 0.1 mm glass beads. Cell debris and beads were removed by centrifugation and RNA purified using a Qiagen RNeasy mini-kit according to the manufacturer’s instructions. Purified RNA was quantified using RNA-specific fluorescent label derivatization (Qubit 2.0; Life Technologies) and quality checked using capillary electrophoresis (Bioanalyzer; Agilent). DNA libraries were produced from RNA samples by TGAC (The Genome Analysis Centre) (Norwich, UK) and the Illumina HiSeq 2000 sequencing platform used to produce 150 bp non-paired end reads.

To map the reads, Bowtie2 software was used with default parameters [29] and filtered with SAMtools to remove non-uniquely mapped reads [30]. A custom Perl script was used to calculate the coverage score for each base position. For absolute expression, these values were used to generate TPM (transcripts per kilobase million) scores for each gene [31]. Normalized differential expression of genes was calculated using DESeq in R [32] using raw coverage scores.

The Rlp4292 genome (NCBI taxonomy ID 936350) was downloaded from the img database [33], along with the Sym plasmid pRL1 from *R. leguminosarum* bv. *viciae* 248 (NCBI taxonomy ID 936136). The RlvA34 genome was electronically reconstituted from that of Rlp4292 by eliminating pRP2 and adding pRL1. Reciprocal blast searches against Rlv3841 and *Rhizobium etli* CFN42 genomes and subsequent parsing using BioPerl improved gene annotation. Homology based searches within Rlp4292 and RlvA34 used usearch [34]. Alignment identity (id) scores were calculated using lalign (www.ch.embnet.org/software/LALIGN_form.html) with default settings. A custom Perl script was used to generate heat map scores for pRP2 and pRL1 gene expression, allowing plasmid maps to be drawn with GenomeVx (http://wolfe.ucd.ie/GenomeVx/) [35].

## Results and Discussion

### Structure of Rlp4292 and RlvA34 genomes

**Fig. 1.**
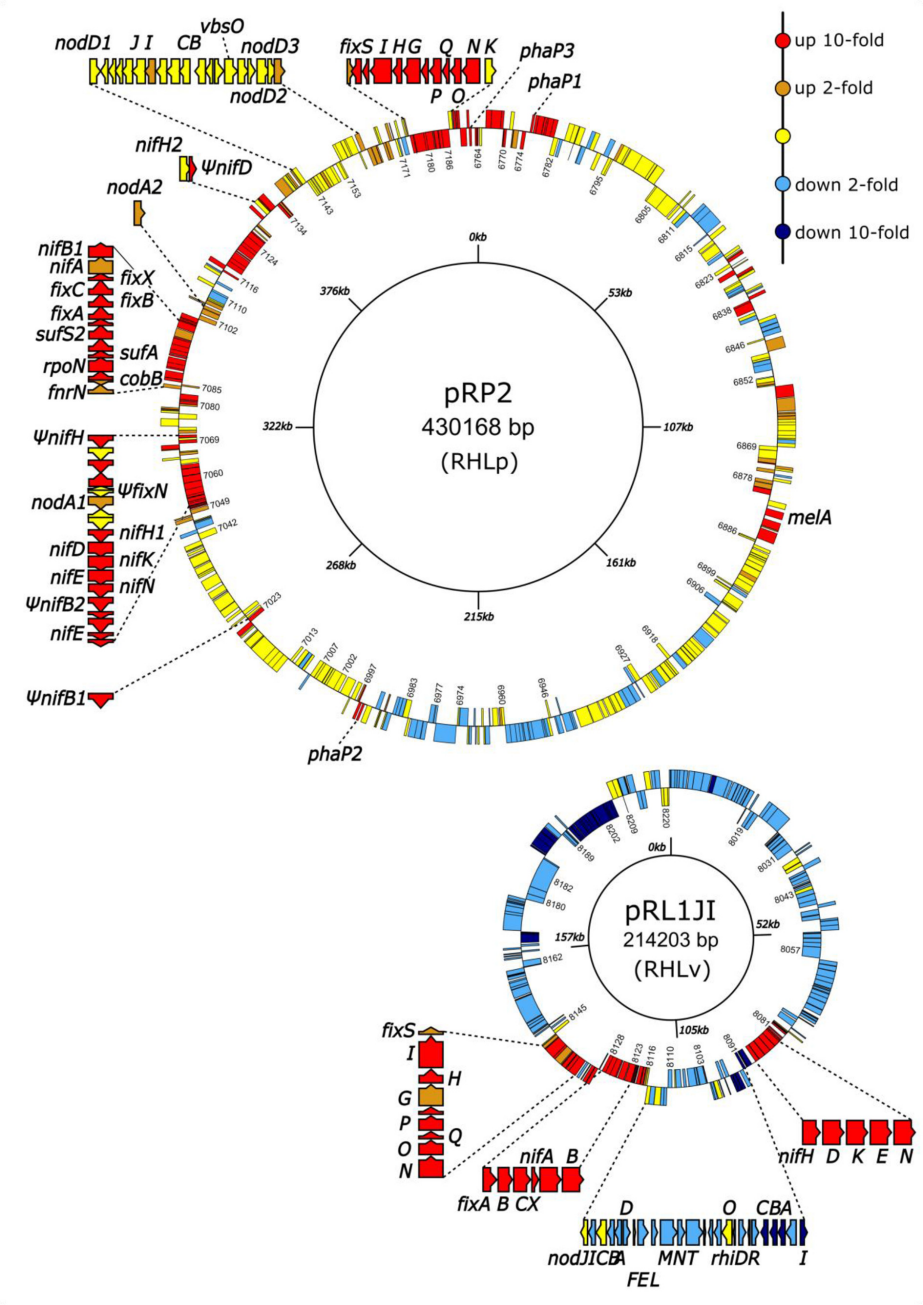
Scale genetic maps of Sym plasmids pRP2 [Rlp4292, 433 genes (*RHLp6764*–*7197*)] and pRL1 [RlvA34, 223 genes (*RHLv8000–8223*)]. Genes are coloured according to differential expression in bean and pea bacteroids. Genes up-regulated >10-fold in bacteroids are red; >2-fold up-regulated, orange; between 2-fold up-regulated and 2-fold down-regulated, yellow; >2-fold down-regulated, light blue; >10-fold down-regulated, dark blue. Exploded regions show the names of genes discussed in the text. The prefix for each gene name is shown in brackets within each replicon and the numbers printed inside the outer ring indicate the gene (e.g. on pRP2: *RHLp7097*, *nifB*). Data for this figure is given in Tables S6 and S8.

The shared genomes of Rlp4292 and RlvA34 comprise four replicons: a 4.7 Mb chromosome (*RHL0001*–*4710*), 1 Mb plasmid A (*RHLa4712–5668*), 594 kb plasmid B (*RHLb6570–6235*) and 562 kb plasmid C (*RHLc6237–6762*) (Fig. S1). The Sym plasmids encode many genes involved in symbiosis and nitrogen fixation (Fig. 1). In Rlp4292, pRP2 is 430 kb with 433 genes *(RHLp6764–7197)*, while in RlvA34 pRL1 is 214 kb with 223 genes (*RHLv8000*–*8223*). Although RlvA34 is a ‘synthetic strain’ made by introducing Sym plasmid pRL1, it is comparable to native strains, e.g. *R. leguminosarum* bv. *viciae* 3841, on pea plants grown without added nitrogen as measured by plant dry weight, acetylene reduction and ^15^N_2_ reduction [21, 36, 37].

Initial annotation of Rlp4292 and RlvA34 [downloaded from the JGI (Joint Genome Institute) database: https://img.jgi.doe.gov/] lacked many conventional gene names (over 2000 gene products labelled as hypothetical). Rlp4292 and RlvA34 share most homology at the nucleotide level with *R. etli* CFN42 [38], whereas genes from pRL1 show highest homology to Sym plasmid pRL10 in the pea-nodulating strain *R. leguminosarum* bv. *viciae* 3841 (Rlv3841) [39]. Annotations from CFN42 and Rlv3841 were used to improve annotation of Rlp4292 and RlvA34. Both pRP2 and pRL1 Sym plasmids encode nitrogen-fixation enzymes in *nif* and *fix* gene clusters. Among pRP2 genes directly involved in symbiotic nitrogen fixation, there are three predicted homologues of *nodD*, and two each of *nodA* and *nifH* (Table S3). The pseudogenes (Ψ) Ψ*fixN* (RHLp7176), Ψ*nifB1* and Ψ*nifB2* (RHLp7023 and RHLp7057), Ψ*nifD* (RHLp7132), and Ψ*nifH* (RLP7071) have inappropriately short predicted gene products (Table S3, Fig. 1) and are not considered further.

### Differential gene expression during growth of free-living Rlp4292 and RlvA34

Gene expression differences in Rlp4292 and RlvA34 grown under identical *in vitro* conditions must be due to their Sym plasmids or to mutations within their genomes; about 97 % of the common genes showed little (<2-fold) or no differences in expression (Table S4). Some common genes (0.3 %) were differentially expressed (>5-fold). In RlvA34, this included the following genes normally induced under iron limitation: *RHLa5120* (*rpoI*); the vicibactin synthesis and uptake cluster *RHLa5121–6* (*vbsLDAGSO*); the *RHL2532–40* genes, including the *hmuPSTU* haemin uptake genes (*RHL2537–40*) [40, 41]; and *RHLa5475-7* encoding a FeCT (iron chelate family uptake) ABC transporter (Table S5. The RNA-Seq data revealed in RlvA34 (but not Rpl4292) a nucleotide (T) deletion in *rirA*, which encodes a repressor of iron-regulated genes [42]. The frameshift would change amino acid lysine 75 to leucine and lead to truncation of 80 amino acids from RirA (161 amino acids). We conclude that mutation of *rirA* caused up-regulation of this regulon of genes in RlvA34.

The only genes expressed more strongly (>5-fold) in Rlp4292 than in RlvA34 were *RLH2777* encoding a putative oxidoreductase and *RHL4467* of unknown function (Table S5). The consistency of expression of shared genes in free-living cultures of Rlp4292 and RlvA34 justifies the validity of comparison of gene expression under symbiotic conditions, with the proviso that the RlvA34 carries a *rirA* mutation causing up-regulation of some iron-related genes.

### Differential expression of Sym plasmid genes in bean and pea bacteroids

**Fig. 2.**
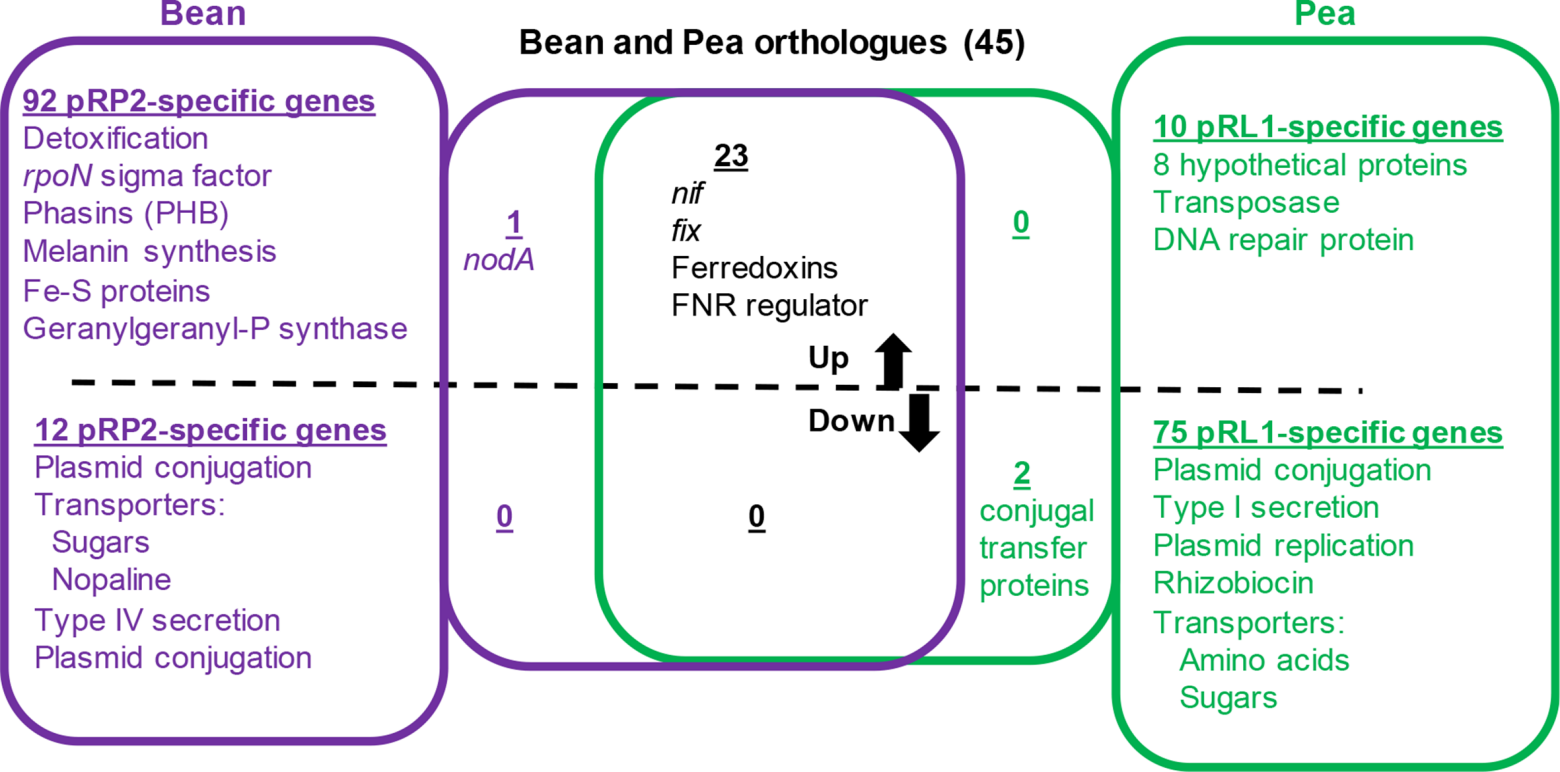
Summary of Sym plasmid genes differentially regulated in bacteroids. Expression data of Sym plasmid genes of pRP2 (433 genes) in bean bacteroids are given in Table S6 and for those of pRL1 (223 genes) in pea bacteroids in Table S8. Data for genes that are common (>80 % id) to both pRP2 and pRL1 (45 genes) are given in Table S9.

**Fig. 3.**
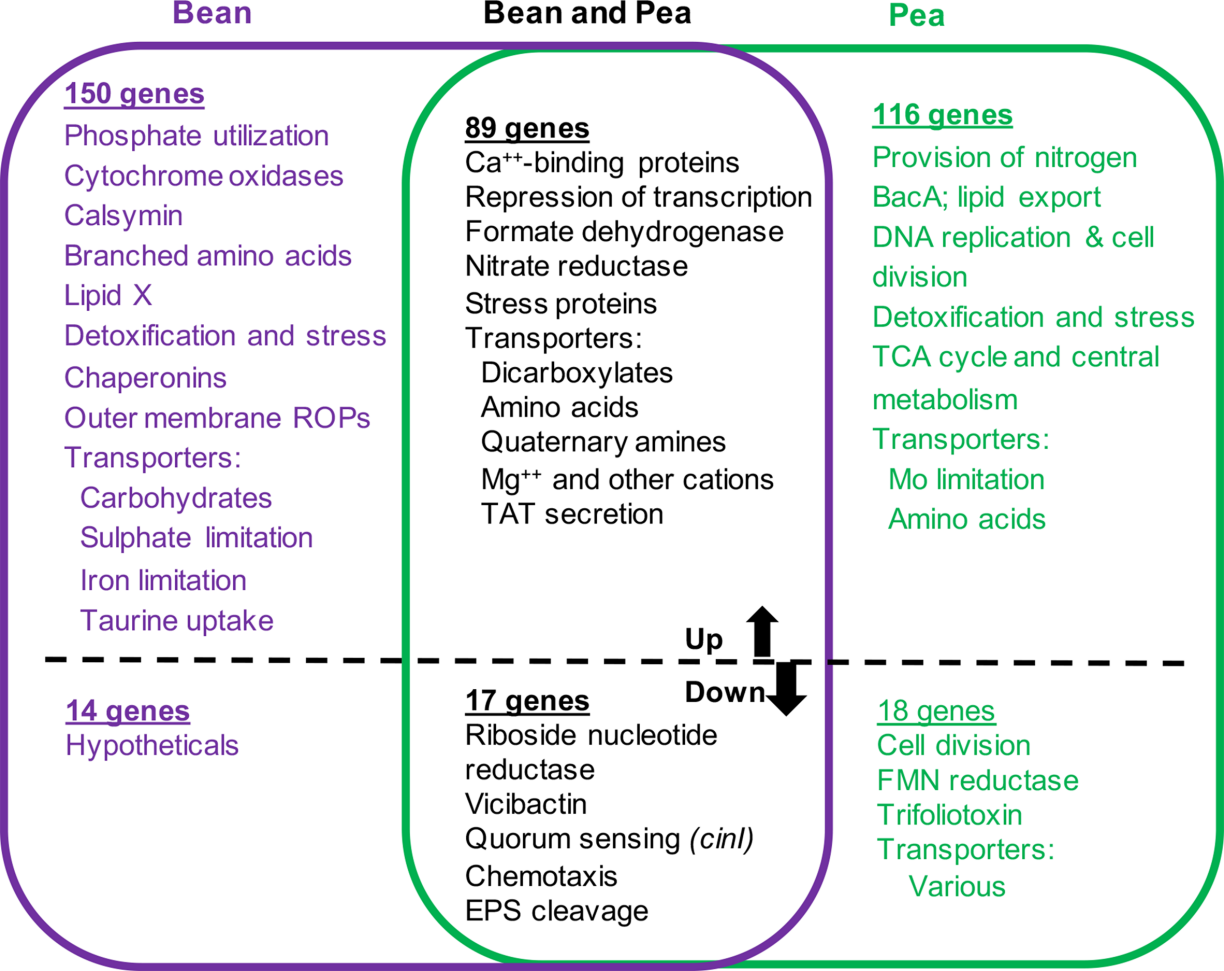
Summary of genes on the shared genome differentially regulated in bacteroids. Genes up-regulated >5-fold in both bean and pea bacteroids (89 genes) are listed in Table S10. Genes up-regulated >5-fold in bean and <2-fold in pea bacteroids (150 genes) are listed in Table S13. Genes up-regulated >5-fold in pea and <2-fold in bean bacteroids (116 genes) are listed in Table S15. Genes down-regulated >5-fold in both bean and pea bacteroids (17 genes) are listed in Table S18. Genes down-regulated >5-fold in bean and >1-fold pea bacteroids (14 genes) are listed in Table S19. Genes down-regulated >5-fold in pea and >1-fold bean bacteroids (18 genes) are listed in Table S20.

RNA sequence reads from Rlp4292 and RlvA34 bacteroids were compared with data from their respective free-living cells to generate normalized values for differential gene expression in bacteroids (Table S6). Fig. S2 shows the proportion of genes, by replicon, that are >2-fold differentially regulated. These data were heavily skewed, with most genes down-regulated, representing the ‘real’ nature of the gene expression in bacteroids, but not allowing accurate comparison of differential gene expression. Therefore, we applied DeSeq normalization to these data to allow calculation of differential gene expression (Table S7). Genes shown as differentially expressed in Table S7 show >5-fold normalized increases or decreases in RlvA34 or Rlp4292 bacteroids relative to the expression in free-living cultures of the same strain; we used this differential in our analyses described below (summarized in Figs 2 and 3). In this section, we will deal with differentially expressed Sym plasmid genes (Fig. 2), and in the next section we will consider the genes on the common genome (Fig. 3).

As the Sym plasmid plays a key role in symbiosis, differential gene expression on plasmids pRP2 (433 genes) and pRL1 (223 genes) is summarized as a heat-map (Fig. 1, showing >2- and >10-fold differential expression, Tables S6 and S8). pRP2 and pRL1 encode 45 gene products showing >80 % amino acid id, and so we considered them as orthologues (summarized in Fig. 2). Table S9 gives their relative expression in bean and pea bacteroids.

#### (i) Expression of common Sym plasmid genes in bean and pea bacteroids

##### Nitrogen-fixation genes

The *nif* genes on pRP2 and pRL1 were up-regulated >10-fold in bacteroids (Fig. 1). The *nifHDKEN* operons (*RHLp7062–58* and *RHLv8088–4*) were up 100- to 460-fold in bean, and 40- to 180-fold in pea bacteroids, respectively (Tables S6, S8 and S9). The *fixABCXnifAB* genes on pRP2 (*RHLp7092–7*) and pRL1 (*RHLv1824–19*) were about 7- to 280-fold and 12- to 40-fold up-regulated, respectively, in bacteroids (Table S9). The *fixGHIS (fixG1H1I1S1*; *RHLp7178–81* and *RHLv8139–42*) and *fixNOQP* (*fixN1O1Q1P1*; *RHLp7186–3* and *RHLv8134–7*) genes were highly up-regulated (Fig. 2, Table S9). The shared genome of Rlp4292 and RlvA34 also contains *fixNOQP* (*fixN3O3Q3P3*; *RHL4126–3*) and *fixSI* (*fixS1I2*; *RHL4119–20)* genes, but these were not induced in bean or pea bacteroids (Table S4), presumably regulation of their expression (differing from that of analogous genes on the Sym plasmid) reflects their upstream control regions and local DNA topology. Also, on the common genome is *fixK1* (*RHLb5914*), encoding an FNR/CRP-family transcriptional regulator; it was about threefold up-regulated in bean but about fourfold down-regulated in pea bacteroids (Table S4).

##### Nodulation (*nod*) genes

The clustered (Fig. 1) *nod* genes on pRL1 [43], *nodABCIJ* (*RHLv8111–15*)*, nodFEL* (*RHLv8109–7*), *nodMNT* (*RHLv8106–4*) and *nodO* (*RHLv8100*) are regulated by the sole copy of NodD (RHLv8110). On pRP2, the *nodABCIJ* genes are more scattered (*nodA1*: *RHLp7065*, *nodA2*; *RHLp7104*, *nodBCSUIJ RHLp7151–*46) (Fig. 1). There are no *nodFEL, nodMNT* and *nodO* genes, but the presence of *nodS* (*RHLp7149*), *nodU* (*RHLp7148*) and *nodZ*, (*RHLp7173*) suggest the Nod factor(s) are similar to that of *R. etli* [44], with *N-*methyl, carbamoyl and fucosyl groups. In RlvA34 bacteroids, *nod* genes were not expressed (Table S8), whereas in bean bacteroids *nodA1* (*RHLp7065*), *nodA2* (*RHLp7104*), *nodD2* (*RHLp7142*), *nodD3* (*RHLp7161*) and *nodU* (*RHLp7148*) were up-regulated about 3- to 9-fold, while other *nod* genes on pRP2 were not. Interestingly, it has been shown that the determinate nodule-forming *Mesorhizobium loti* requires expression of specific *nodD* genes at different stages through nodule formation on *Lotus japonicus,* and it is thought that these form additional checkpoints for the infection process [45]. For indeterminate nodules where there is only a single *nodD*, and where *nod* gene expression is strongly up-regulated in the pea rhizosphere and infection threads [46], but rapidly ceases following release from infection threads [47], the observed low level of *nod* gene expression in bacteroids means there must be very little contamination of these RlvA34 pea bacteroids with infection threads or extracellular rhizobia. Common Sym plasmid genes >5× down-regulated in pea include *RHLp6783*/*RHv8206* (*traI*) and *RHLp6974*/*RHLv8171* (*traD*) involved in plasmid conjugation, which are known to be induced in free-living cultures [48].

#### (ii) pRP2 genes differentially regulated in bean bacteroids

In Rlp4292 bacteroids, 27 % of the Sym plasmid pRP2 genes were >5-fold up-regulated and 15 % were >5-fold down-regulated (Table S7). As pRP2 contains 210 more genes than pRL1 (Fig. S1), many pRP2-unique genes with no pRL1 orthologue are differentially regulated in bean bacteroids (summarized in Fig. 2).

##### pRP2 genes up-regulated in bean bacteroids

Many up-regulated genes are predicted to be involved with detoxification or export of unwanted chemicals/toxins, presumably reflecting the environment within bean nodules (Table S6). Genes *RHLp7122–6* and *RHLp7083* (up-regulated 60- to 150-fold) encode five cytochrome P450 monooxygenase proteins and an oxidoreductase (Table S6). *RHLp7189–97* (up-regulated 10- to 100-fold) encode genes for a putative ferredoxin and two copper-containing oxidases (Fig. 1, Table S6). Several of these predicted monooxygenases, oxido-reductases and oxidases could target specific nodule compounds made in bean but not pea nodules. In view of the observation that peas make NCR defensin-like peptides but beans and other determinate nodules do not [6], secondary antimicrobial metabolites may be of importance in bean to reduce infection by other bacteria (cheaters) that do not contribute to symbiotic nitrogen fixation.

##### Nitrogen-responsive sigma factor RpoN

In rhizobia, the sigma factor σ^54^ (RpoN) regulates the expression of nitrogen fixation (*nif/fix*), nitrite assimilation (*nir*) and C4-dicarboxylate transport (*dct*) genes. Some rhizobia have a single *rpoN* gene, but others, including *B. japonicum* [49] and *R. etli* CFN 42 [50], have two, one being induced at low oxygen levels during symbiosis or during free-living growth, and the other being negatively auto-regulated [49, 50]. In Rlp4292, *rpoN2* on pRP2 (*RHLp7087*) was up-regulated about 160-fold in bean bacteroids (Table S8), whereas the chromosomal *rpoN* (encoding RHL4002, 55 % id to RHLp7087) was about 2-fold down-regulated in bean bacteroids. RlvA34 has only the chromosomal *rpoN* (*RHL4002*), which was up-regulated about eightfold in bacteroids.

##### Phasin genes

The most highly up-regulated pRP2 genes (increased more than 500–600-fold) were *RHLp6775, RHLp6995* and *RHLp7194,* encoding phasins (95–98 % id) (Table S6). These non-catalytic proteins coat polyhydroxyalkanoate (PHA) granules, preventing formation of large masses of PHA [51]. Their role is probably to coat the extensive granules of polyhydroxybutyrate (PHB, a form of PHA) found in Rlv4292 bacteroids [37]. Although RlvA34 produces PHB in pea nodule infection threads, PHB granules are not seen in mature pea bacteroids [37] and pRL1 lacks the phasin genes found on pRP2. PHB synthase (*phaC*, *RHL1006*) was about threefold up-regulated in bean (Rlp4292) but not in pea (RlvA34) bacteroids (Table S4). The formation of PHB and/or lipid storage polymers enables recycling of CoA from acetyl-CoA.

##### Melanin

Production of melanin in Rlp4292 requires pRP2-encoded MelA (RHLp6882) [52], which oxidises tyrosine immediately prior to its polymerization into melanin. In bean nodules, *melA* (*RHLp6882*), which is co-regulated with *fix* genes [53], was up-regulated about 300-fold (Table S6). Melanin can trap free radicals that are produced during nitrogen fixation, possibly reflecting a different redox environment within bean and pea bacteroids.

##### Fe-S proteins

Genes encoding a Fe-S cluster assembly protein (RHLp7088) and a 4Fe-4S binding domain protein (RHLp6777) were up-regulated 40- and 100-fold, respectively, in bean nodules, and could be involved in electron transfer during nitrogen fixation or to other proteins, such as cytochrome P450s.

##### pRP2 genes down–regulated in bean bacteroids

Although about 45 % of pRP2 genes were not differentially expressed >2-fold in bacteroids (yellow in Fig. 1, Table S6), many genes were down-regulated (>2-fold) in bean bacteroids (light blue in Fig. 1, Table S6), including genes encoding plasmid conjugation (*RHLp6940–5* and *RHLp6969–81*), putative ABC transport systems for sugars (*RHLp6928–32*) and nopaline (*RHLp6983–7*), components of a type IV secretion system (*RHLp6948–59*) and transposase proteins (*RHLp7043–5, RHLp7110–1* and *RHLp7172–4*) (Table S6).

#### (iii) pRL1 genes differentially regulated in pea bacteroids

In RlvA34 pea bacteroids, 15 % of pRL1 genes were up-regulated >5-fold, whereas 34 % were down-regulated >5-fold (the highest proportion of any replicon) (Table S7). In addition to the aforementioned *nif* (*RHLv8080–8*)*, fixABCX nifAB* (*RHLv8118–28*) and the *RHLv8134–8142 fix* genes (shown in red in Fig. 1), other genes up-regulated are *RHLv8117*–*20* (about 5- to 30-fold), RHLv8130*–*1 (5- to 30-fold), *RHLv8083* (about 20-fold), *RHLv8121* (about 30-fold) and *RHLv8138* (about 30-fold) (Table S8). The functions of the products of several of these genes are not known.

Among the 75 % of down-regulated genes are *RHLv8180–2* and *RHLv8185–7* encoding putative rhizobiocins and possibly their type I export systems and other ABC transporters; *RHLv8035–9* [PAAT (polar amino acid transport) family], *RHLv8059–62* [CUT2 (carbohydrate uptake transporter 2) family] and *RHLv8066–9* [CUT1 (carbohydrate uptake transporter 1) family] (Figs 1 and 2, Table S8).

### Differential expression of the common genome in mature bacteroids

Differential expression of the common genome (i.e. everything other than the Sym plasmids) is summarized in Fig. 3 and Table S7 shows the numbers of genes differentially expressed >5-fold, by replicon, for bean and pea bacteroids. On each replicon, similar numbers of genes were up-regulated >5-fold in both bean and pea bacteroids (between 7 % on plasmid B and 2 % on plasmid A), except for plasmid C with 27 genes up-regulated (5 %) in bean compared to only 10 (2 %) in pea bacteroids. For down-regulated genes in the two bacteroid types, similar values were seen for the chromosome and plasmid B (1–2 %), but for plasmids A and C, a slightly higher percentage is down-regulated in bean (5–6 %) than in pea (1–3 %) (Table S7).

#### (i) Shared genes up-regulated in both bean and pea bacteroids

Pathways important for symbioses in both determinate and indeterminate nodules can be deduced from Table S10 showing the 89 genes >5-fold up-regulated in both bean and pea bacteroids. For comparison, this table includes data with likely orthologues in Rlv3841 from pea nodules [13].

##### Calcium-binding proteins

*RHL1101* and *RHLb6093* encoding EF-hand calcium (Ca)-binding proteins were strongly up-regulated in bean and pea bacteroids (Table S10). Similar rhizobial proteins have two predicted EF-hand domains [50], one extending outside the cell. Ca-binding proteins have several roles including Ca homeostasis, signalling between bacteroid/plant and may be expressed as a result of nitrogen-starvation or stress (for reviews see [54, 55]).

##### Repression of transcription

DksA proteins bind to RNA polymerase and mediate the stringent response induced on nutrient limitation. *RHLb6067* (*dksA1*) and *RHL1099* (*dksA2*) were up-regulated about 7- to 40-fold. Mutation of *dksA* in *S. meliloti* reduced symbiotic nitrogen fixation in alfalfa nodules [56].

##### Formate dehydrogenase

Genes encoding formate dehydrogenase subunits and associated proteins were up-regulated 5- to 20-fold (*RHL3212–16; fdhDA2BG*) (Table S10), and 110- and 20-fold (*RHLp7098*/*RHLv8122; fdsA3*) (Table S9), in bean and pea bacteroids, respectively. An exception is formate dehydrogenase subunit A, *RHL3088* (*fdsA1*), which was not differentially regulated (Table S4).

##### Stress proteins

Nine genes encoding stress proteins were up-regulated in both bean and pea bacteroids (Table S10, Riley code 1.6.1). *RHLb6081* (*hspF*) was about 400- and 70-fold up-regulated in bean and pea bacteroids, respectively; *RHLb6065* was about 30- and 40-fold up-regulated in pea and bean, respectively; *RHL1259* was 12- and 20-fold up-regulated in bean and pea bacteroids, respectively.

##### Transport systems

Transport system genes are often induced in response to the transported solute; thus, giving an indication of the chemical environment. Twenty genes whose products are predicted to be involved in transport across membranes were up-regulated >5-fold in both bean and pea bacteroids (Table S11, selecting Riley code 1.5.x). To ensure the most robust data, we chose to examine transporters that were not only up-regulated, but also highly expressed (>200 000 reads); 15 genes fulfilled these criteria (Table S12, Fig. 3) and are described below.

##### Bacteroids are fuelled by C4-dicarboxylates

The C4-dicarboxylate transporter *RHL2260* (*dctA*) was up-regulated about 14- and 5-fold in bean and pea bacteroids, respectively (Table S12) (appearing as bean-specific in Fig. 3 due to falling just below the 5-fold cut-off). It was the most highly expressed bacteroid transporter with >8×10^6^ and >3×10^6^ reads, respectively (Table S12); it is also the most highly expressed transporter in succinate-grown free-living RlvA34 and second most expressed in succinate-grown free-living Rlp4292 (Table S4). Therefore, increased expression of *dctA* in bacteroids, relative to even succinate-grown cultures, illustrates the importance of dicarboxylates as a carbon source in nodules.

##### Amino acids

Four genes encoding components of the Bra (branched-chain amino-acids) transporter [ABC HAAT (hydrophobic amino acid transporter) family] were up-regulated >5-fold in bacteroids. Although Fig. 3 shows *RHL2378* (*braC3*) and RHL2589 (*braC*) specifically up-regulated in bean bacteroids and *RHL2591* (*braG*) and *RHL2592* (*braF*) specifically in pea, closer inspection reveals that all genes are up-regulated by >2-fold in both bacteroid types (Table S12). Pea and bean bacteroids require the plant to supply branched-chain amino acids isoleucine and valine to allow bacteroid development [57]. The broad-specificity amino acid transporters Aap and Bra [57, 58] are essential for bacteroid branched-chain amino acid uptake and normal nitrogen fixation in Rlv3841 [36, 59]. The PAAT family ABC transporter Aap is unusual in that it transports a wide-range of substrates [60, 61]. Aap components encoded by *RHL1044-6* (*aapPMQ*) were up-regulated about 4- to 7-fold in both bean and pea bacteroids (Table S12). The importance of both transport systems in nodules is illustrated by the observation that although strains Rlv3841 and Rlp4292 mutated in both Aap and Bra form nodules on peas and beans, respectively, they fix nitrogen at only about 30 % of the wild-type rate [21, 62, 63]. These data are indicative that supply of these amino acids to pea and bean bacteroids is similar.

##### Quaternary amines and other nitrogenous compounds

The ABC QAT (quaternary amine transporter) family transports quaternary amines, such as histidine and choline. In both bean and pea bacteroids, *RHL2371* and *RHLa5629* [which encode solute binding proteins (SBPs) GbcX (QatX1) and QatX3, respectively] were up (about 7- to 30-fold), as were the contiguous genes *RHL2372–3* (*qatW1V1*) (3- to 9-fold) suggesting that choline and/or glycine betaine are used by both bean and pea bacteroids. Mutation of the QAT encoded by *RL3533–5* (*gbcXWV)* inhibited uptake of choline and glycine betaine, and the residual low transport of glycine betaine was attributed to the Qat3 system (pRL120514*–*6) [64]. RHLa5629 shows 96 % id with pRL120516 and it may be that it forms part of a second glycine betaine transport system. *RHL2564* encoding an SBP of an ABC transporter of the NitT (nitrate/nitrite/cyanate transporter) family was up-regulated in bacteroids of both Rlp4292 (about 13-fold) and RlvA34 (about 6-fold) (Fig. 3); the orthologous gene in *S. meliloti* (SMc01827) is induced by uracil and uridine [65].

##### Magnesium and other cations

Expression of *mgtE* (*RHL0406*), encoding a Mg^++^ transporter was up-regulated about 18-fold in bean and about 4-fold in RlvA34 pea bacteroids (the >5-fold cut-off making it appear bean-specific in Fig. 3). Cation-transporting ATPase proteins encoded by *RHLb6066* and *RHLb6068* were up-regulated about 20- to 30-fold and about 6- to 7-fold in bacteroids of Rlp4292 and RlvA34, respectively. MbfA (RHL3841), a putative rubrerythrin (contains ferritin fold) transmembrane protein, is related to proteins involved in iron and manganese transport (CCC1-like family), and *RHL3841* was up-regulated about 23-fold in beans and about 5-fold in peas (Table S12, Fig. 3).

##### Tat secretion

The Tat protein exporter encoded by *RHL0954* (*tatA*) and *RHL0955* (*tatB*) was up-regulated about sixfold in RlvA34 pea bacteroids (Fig. 3) and about threefold in bean bacteroids (Table S12). This transporter secretes cell wall amidases needed for rhizobial-wall integrity and nodule infection; it also exports the periplasmic Rieske electron transport protein, required for bacteroid respiration [66, 67].

##### Role of plasmid B in symbiosis

Within a cluster of just over 30 genes encoded on plasmid B (*RHLb6065–6098*), 24 are up-regulated >5-fold in both bean and pea bacteroids (Table S10). Although several genes in this cluster are related to stress responses: e.g. *RHLb6065* and *RHLb6072* encode universal stress proteins; *RHLb6081* encodes a small heat shock protein, HspF (see section above); *RHLb6066* and *RHLb6068* encode cation transporters; and *RHLb6098* encodes FeuP, part of a two-component sensor regulator (Table S10). The significance of this clustering of genes is not known.

#### (ii) Shared genes up-regulated only in bean bacteroids

One hundred and fifty genes were >5-fold up-regulated in Rlp4292 bean bacteroids and <2-fold up-regulated in RlvA34 from pea nodules (Table S13, Fig. 3). In this section, we deal with supply of P, S, Fe, Ca, C, N and O_2_, and then predicted stresses.

##### Bean bacteroids are phosphate-limited

Several phosphate-related genes were up-regulated in bean but not pea bacteroids, including: *RHL3750–52*, *RHL3755* (about 5- to 30-fold) encoding components of the PhoT (phosphate/phosphonate transporter) family ABC system; *RHL0576* and *RHL3757–9* (about 5- and 210-fold) encoding four putative phosphonate utilization proteins; *RHL3549* (about 60-fold) encoding an alkaline phosphatase PhoA; and *RHLb6029* (about 110-fold) encoding an acid phosphatase, possibly involved in glycerolipid metabolism (Table S13). Under phosphate limitation, *S. meliloti* induces the *btaAB* genes to make phosphate-free lipids from diacylglycerol (DAG) [68]. In bean (but not pea) bacteroids, *btaA* and *btaB* (*RHL2133–4*) were up-regulated about 25- and 10-fold, respectively. Expression of *btaAB* is induced by PhoB–PhoU sensor-regulator proteins that are encoded by *RHL4136–7* (up about 13-fold in bean). The contiguous genes (*RHL4132–5)* encode a PhoT ABC transporter for uptake of phosphate and were up-regulated 15- to 75-fold in bean bacteroids (Table S13). Taken together, these data suggest that Rlp4292 bacteroids are phosphate-limited compared to RlvA34 bacteroids. In *Sinorhizobium* NGR234, genes encoding phosphate uptake systems and *btaAB* were up-regulated in both determinate and indeterminate nodules [18], so nodule bacteroids may be phosphate-limited in some legumes but not others.

##### Bean bacteroids are sulphate-limited

**Fig. 4.**
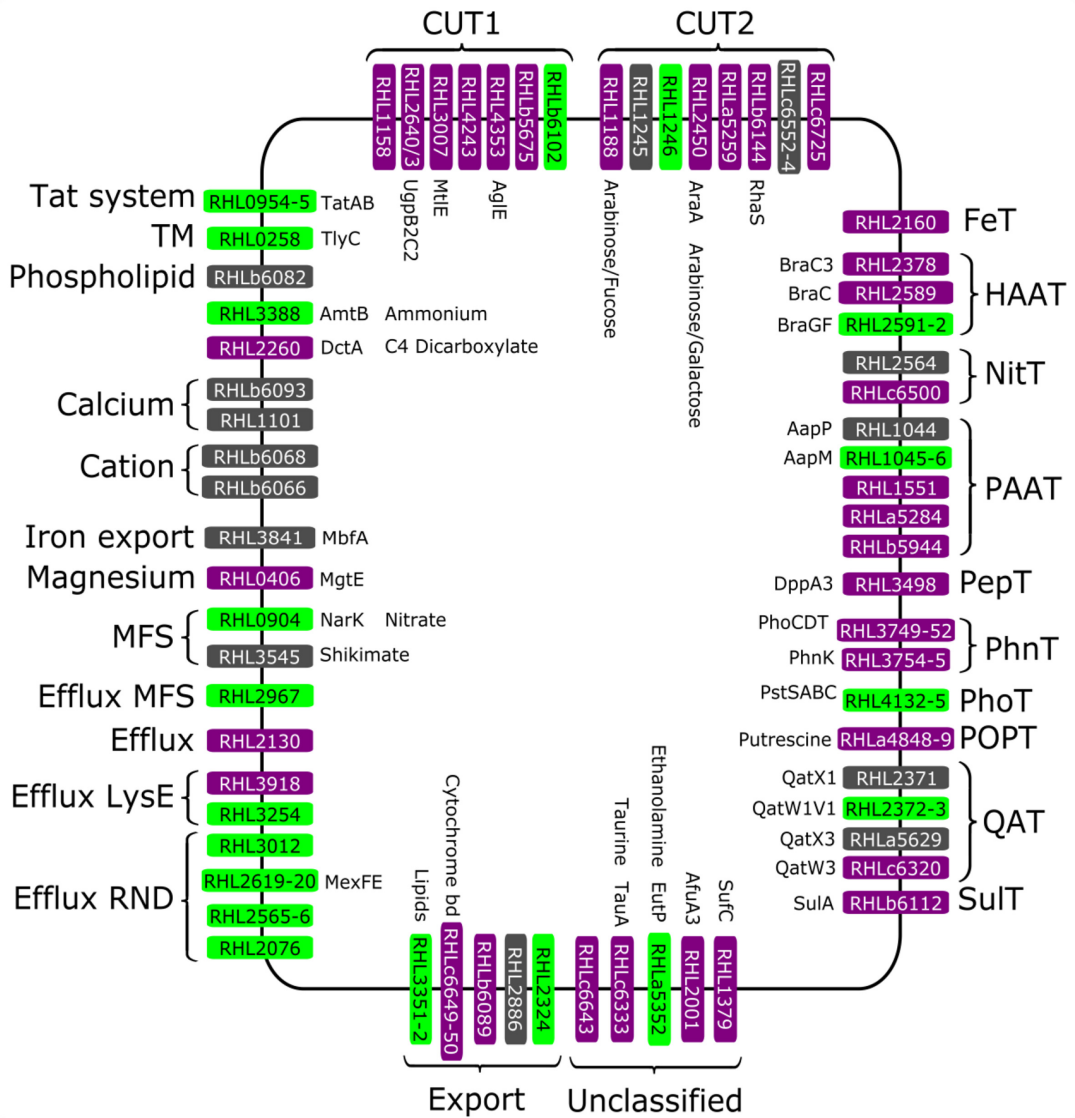
Transporter systems whose genes are up-regulated in Rlp4292 and RlvA34 bacteroids. Common nodule-specific transporters (up-regulated >5-fold in nodules of both strains) are shown in black, those specific to bean (up-regulated >5-fold in bean bacteroids and <5-fold in pea bacteroids) are shown in purple and those specific to pea (up-regulated >5-fold in pea bacteroids and <5-fold in bean bacteroids) are shown in green. In addition to being up-regulated in bacteroids, all genes are also highly expressed (>200 000 reads). Data for this figure is given in Table S12.

*RHLb6112* encoding the sulphur transport ABC component SulA was up-regulated about sixfold in bean and about sixfold down-regulated in pea bacteroids (Table S12, Fig. 4). The *sulA* (*SMb21133*) orthologue in *S. meliloti* is induced by sulphate limitation [65]. *RHLc6500,* a NitT family transporter that probably encodes an aliphatic sulphonate ABC transporter SBP, was up-regulated about sevenfold in bean bacteroids but down-regulated about threefold in pea (Fig. 4). This suggests that bean bacteroids are sulphate-limited, while those of pea are not.

##### Bean bacteroids are iron-limited

Several uptake systems associated with iron limitation are up-regulated in bean but not pea bacteroids; none of these genes (described below) is among those up-regulated in free-living RlvA34 compared with Rlv4292 (Table S5). This means that there are at least two sets of transcriptional responses to iron. The ABC transporter FeT [Fe (III) transport] family gene *RHL2160* and *RHL2001* (*afuA3*) were strongly up-regulated (about 20- and 40-fold, respectively) in bean but not pea bacteroids (Table S12, Fig. 4). *RHL1379* (*sufC*), involved in [Fe-S] cluster assembly, was up-regulated about sixfold in bean and fourfold in pea bacteroids (Table S12). The PepT family dipeptide transporter, *RHL3498* (*dppA3*), was up-regulated about sixfold in bean and threefold in pea bacteroids (Table S12). Mutation of the rhizobial *dpp* operon reduces uptake of the haem precursor δ-aminolevulinic acid [69]. In *Escherichia coli*, the Dpp transport system transports iron via haem [70].

##### Calcium

In addition to the Ca-binding proteins up-regulated in both bean and pea bacteroids (see above), *RHLa5297* (*casA*) encoding the exported Ca-binding protein calsymin, was about 50-fold induced in bean but not pea bacteroids (Table S13). *R. etli* CasA (85 % id with RHLa5297) is important for the symbiosis with *Phaseolus*, because a *casA* mutation affected bacteroid development and decreased nitrogen fixation [55].

##### Transport systems up-regulated only in bean bacteroids

Fifty-six genes encoding components of solute uptake systems were up-regulated >5-fold in Rlp4292 bean bacteroids and <5-fold in RlvA34 bacteroids (Table S14, Riley code 1.5.0–1.5.4) and 44 of these were highly expressed (Table S12, Fig. 3).

##### Diverse carbohydrates available in bean nodules

Expression of carbohydrate uptake transporters (CUTs) gives an insight into sugars available in nodules. There are two sub-classes of these ABC transporters: CUT1, transporting di- and oligo-saccharides; and CUT2, which generally transports monosaccharides. Eight genes encoding CUT1 components were up-regulated in bacteroids, seven of these in bean but not pea bacteroids, identifying six different CUT1 transporters (Fig. 4). The SBPs RHLb5675 and RHL3007, respectively, probably bind galactosamine and mannitol, which induce the orthologous genes in *S. meliloti* [65]. *RHL4353*, annotated as alpha-glucoside transporter (*aglE)*, and *RHL2640* and *RHL2643*, annotated as glycerol-3-phosphate transporter components (ATP-binding component and SBP, *ugpC2* and *ugpB2*, respectively), were up-regulated about 80-fold in bean bacteroids (Table S12, Fig. 4).

Ten genes encoding CUT2 components of eight transport systems were up-regulated >5-fold in bean bacteroids. Two systems were up-regulated in both bean and pea bacteroids; *RHLc6553–4* (about 10- to 50-fold) and *RHL1245* (about 10- to 25-fold). The solutes transported by these systems are unknown. Five CUT2 SBP genes were up-regulated in bean but not pea bacteroids: *RHL1188* (about 60-fold), likely binds fucose (its orthologue is induced by fucose in *S. meliloti* [65]); *RHL2450*, likely to bind arabinose and/or galactose (as does its orthologue in Rlv3841 [46]); *RHLa5259* (unknown solute); *RHLb6144*, encoding an SBP involved in competition for nodulation (annotated as a rhamnose transporter); and *RHLc6725*, likely solute arabinogalactan/related compound (from homology with genes in Rlv3841) [46] (Fig. 4). We conclude that bean bacteroids contain a variety of sugars [e.g. galactosamine, mannitol, alpha-glucoside(s), glycerol-3-phosphate, fucose, arabinose/galactose, rhamnose, arabinogalactan] absent from pea bacteroids.

##### Taurine in bean nodules

*RHLc6333* (*tauA*), which encodes a predicted taurine uptake component, was up-regulated about sixfold in bean but down-regulated about eightfold in pea bacteroids (Fig. 4). This is indicative that there is taurine in bean but not pea nodules because RHLc6333 shows 40 % id with the TauT family SBP SMb21526 from *S. meliloti*, which is induced by taurine [65] and forms the basis of a taurine-inducible expression system in rhizobia [71].

##### Other transporters induced in bean nodules

Among the several putative POPT (polyamines, opines and phosphonate) family genes in the shared genome, only *RHLa4848–9* encoding a putative POPT transporter was up-regulated 10- to 40-fold in bean but not RlvA34 pea bacteroids (Table S12, Fig. 4). Three PAAT predicted amino-acid SBP genes (*RHL1551*, *RHLa5284* and *RHLb5944)* were up-regulated 5- to 10-fold in bean but not pea bacteroids (Fig. 4). A predicted QAT system (*RHLc6320)* was up-regulated about 160-fold in bean but not pea bacteroids. In all these cases it is likely that the unidentified solutes are present in bean but not pea nodules.

##### Cytochrome oxidases

Genes *RHLc6648–53,* encoding a transmembrane cytochrome *d* ubiquinol oxidase subunit I, a cytochrome *bd*-II oxidase subunit II, an ABC transporter system related to cytochrome *bd* export and a MarR family regulator of gene expression, were all more strongly up-regulated in bean bacteroids (about 12- to 90-fold, Table S4) than in pea bacteroids (about 2- to 5-fold) (Table S4). Genes encoding cytochrome c oxidase subunits RHL4623*–*4 (CtaC1 and CtaC2) were up-regulated about sixfold specifically in bean bacteroids (Table S13). Cytochrome *cbb3* (encoded by the *fixNOQP* operon) is the high affinity oxidase essential for nitrogen fixation [72], but the expression of other respiratory pathways terminated by oxidases with a lower affinity for oxygen could be indicative that there may be a higher free oxygen level in (parts of) bean nodules.

##### Lipid X

Lipid A is the primary lipid in the outer layer of the Gram-negative bacterial membrane and acts as an anchor for lipopolysaccharide [73]. Synthesis of lipid A involves nine enzymes, one of which, LpxH, cleaves UDP-2,3-diacylglucosamine to 2,3-diacylglucosamine 1-phosphate (lipid X) and uracil-monophosphate (UMP) [74]. *lpxH* (*RHL1469*) was up-regulated about 70-fold in bean but not pea bacteroids. Genes encoding the other eight Lpx enzymes were not up-regulated in Rlp4292 bacteroids (Table S4). Lipid A oxidase *RHL4482* (*lpxQ*) was about sevenfold up-regulated in bean but not pea bacteroids (Table S13).

##### Detoxification and stress

Nine genes encoding stress proteins were elevated specifically in bean bacteroids (Table S13, Riley code 1.6.x). *RHL3015*, encoding the osmotically-induced OsmC, and *RHL1758* and *RHLa5426,* encoding two cold shock proteins, were up-regulated about 10-fold in bean but not pea bacteroids. Glutathione *S*-transferases (GSTs) are diverse enzymes involved in detoxification of oxidative stressors, antimicrobial agents and metabolic intermediates in bacteria. Three predicted GSTs, *RHL0225*, *RHL346* and *RHL3926,* were up-regulated about 10-fold in bean but not pea bacteroids (Table S13). Export is another way of removing toxic compounds; *RHL0518*, *RHL2130* and *RHL3918* encode efflux systems that are up-regulated about 5- to 8-fold in bean but not pea bacteroids (Table S13, Riley code 1.5.5). These data could be indicative that rhizobia in bean nodules are more stressed by metabolites than rhizobia in pea nodules. Chaperonins are also often induced in response to stress [75] *RH2128*, encoding a DnaJ family protein, and *RHL4498–9,* predicted to encode the chaperonins Cpn60 and Cpn10, were about sevenfold up-regulated in bean but not pea bacteroids (Table S13).

##### Outer membrane ROPs

Bacterial outer membranes and their proteins are crucial for cell–cell and cell–environment interactions. In *S. meliloti*, a putative transmembrane β-barrel porin, RopA1, is essential for viability, despite the presence of *ropA2*, encoding a close homologue [76]. The Rlp4292 and RlvA34 share four homologues of RopA (RHL0466, RHL1573, RHL2878 and RHLb6113), each with about 50 % id with RopA1 or RopA2 from *S. meliloti*. Three of these genes, which we named *ropA1* (*RHL0466*)*, ropA3* (*RHL2878*) and *ropA4* (*RHLb6113*), were up-regulated (about 25-, 90- and 10-fold, respectively) in bean but not pea bacteroids (Table S4). Autoaggregation proteins RapA2 (*RHLc6544)* and RapB3 *(RHL2729)* were up-regulated 40- and 12-fold in bean but not pea bacteroids (Table S13). Possibly the presence of multiple bacteroids within one symbiosome as observed in bean but not pea nodules influences expression of genes that affect cell–cell interactions.

#### (iii) Shared genes up-regulated in pea but not bean bacteroids

Table S15 shows the 116 genes that are >5-fold up-regulated in RlvA34 pea bacteroids and <2-fold up-regulated in Rlp4292 from bean nodules. These data are presented together with microarray data for those Rlv3841 genes showing >80 % amino acid id to those of Rlp4292/RlvA34 [13]. The pattern of up-regulated genes is different from that in bean, suggesting limitation of N and Mo and different gene induction associated with C metabolism and bacteroid development. Forty genes encoding components of solute uptake systems were up-regulated >5-fold in RlvA34 pea bacteroids and <5-fold in Rlp4292 bean bacteroids (Table S16, Riley code 1.5.0–1.5.4), with 26 of them highly expressed (Table S12, Fig. 4)

##### Provision of nitrogen for pea bacteroids

**Fig. 5.**
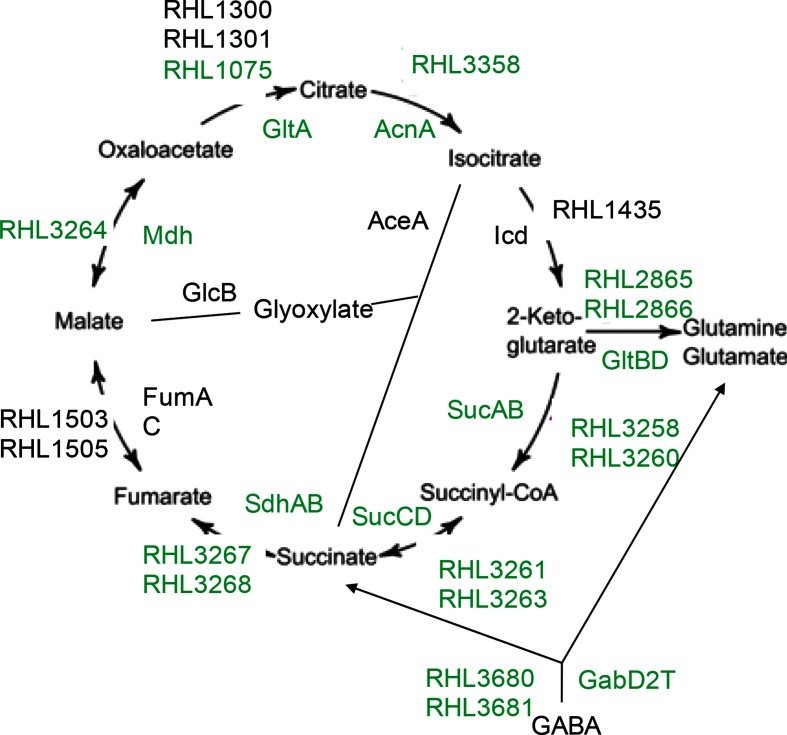
TCA cycle and associated metabolic pathways. Enzymes whose genes are up-regulated >10-fold in RlvA34 pea bacteroids are shown in green. Data for this figure are given in Table S17.

Several genes related to nitrogen utilization were up-regulated in pea but not bean bacteroids. These include *gltDB* (*RHL2865–6*) encoding glutamate oxoglutarate amido transferase (GOGAT), which were up-regulated about 20-fold in RlvA34 (but were unchanged in Rlp4292 bean bacteroids; Fig. 5, Table S17), the ammonium transporter gene *amtB* (*RHL3388*) (Fig. 3), and its cognate regulator *glnK* (*RHL3389*) (about sevenfold and ninefold up-regulated, respectively). Genes (probably an operon) encoding the nitrate reductases/subunits, NasA (RHL0901), NirD (RHL0902)and NasD (RHL090*3*), and NarK (RHL0904), a major facilitator subfamily (MFS) nitrate transport protein (Fig. 3), were up-regulated 10–30-fold (Table S4). Legume nitrate transporter, nitrate reductase and nitrite reductase genes are also up-regulated in nodules [77], especially in the nitrogen-fixation zone [23]. Surprisingly, therefore, it seems that at least under some conditions there must be significant amounts of nitrate in nodules of some legumes.

Phenylalanine could also be a source of N in pea bacteroids because *RHL0852* (*phhA*), encoding a phenylalanine-4-hydroxylase, was up-regulated about 6-fold in pea but not bean bacteroids (it is also up-regulated about 40-fold in the pea rhizosphere; Table S4). Phenylalanine is a precursor of lignin synthesis and may be available in pea nodules due to cell wall synthesis being maintained in mature indeterminate (pea) but not determinate (bean) nodules.

##### Pea bacteroids appear molybdate-limited

The gene encoding SBP ModA (*RHL3521*) was up-regulated about eightfold in pea but not bean bacteroids (Table S16) (this gene failed to meet the criterion for high expression and does not appear in Table S12 or on Fig. 4). Although in Rlv3841, expression of a molybdate transporter (MolT family of ABC transporters) encoded by *modABC* was also induced under low sulphate [78], the fact that ABC sulphur transporter SulT family gene *RHLb6112* (*sulA*) was down-regulated (about 10-fold) in pea bacteroids of RlvA34 and Rlv3841 (pRL110374, 97 % id) (Table S12) suggests that although RlvA34 bacteroids are molybdate-limited, they have sufficient sulphate within pea nodules.

##### Bacteroid development

NCR peptides secreted by the plant [11] affect bacteroid development in peas but not beans, and the resulting differences in bacteroid differentiation are likely to cause differences in bean and pea bacteroid gene expression. Several lipid biosynthesis and metabolism genes were slightly up-regulated (about 2- to 3-fold) in pea, but not bean bacteroids. These include genes encoding the enzymes of the lipid A biosynthetic pathway: *lpxB* (*RHL1074*), *lpxD* (*RHL1070*) and *lpxK* (*RHL4516*), lipid metabolic enzyme (*RHL2015*), an ABC family lipid exporter (*RHL3351–2*). An acyl carrier protein (*RHLc6550*) was up-regulated about 10-fold in pea bacteroids and about 4-fold in bean bacteroids (Tables S4 and S13). The level of induction of most of these genes was modest and not particularly different from those seen in bean bacteroids. This may reflect the fact that changes attributable to NCR peptides have already occurred by the time nodules have matured, a conclusion reached with RNA from Rlv3841 bacteroids of 7, 15, 21 and 28 days post-inoculation analysed using microarrays [13].

Some genes encoding DNA replication functions were up-regulated in pea but not bean bacteroids, including *gyrB1* (*RHL3590*), (Table S15), *dnaX* (*RHL3716*) and *dnaG* (*RHL2242*) up-regulated about sixfold, sixfold and fourfold, respectively (Table S4). This would be consistent with increased DNA endoreduplication in pea bacteroids [11].

##### Detoxification and stress

A predicted salicylate hydrolase gene (*RHLa4811)* was about 12-fold up-regulated in RlvA34 pea bacteroids. A putative arsenate reductase gene (*RHL4205)* was up-regulated about sevenfold in RlvA34 (Table S15). The heat shock protein RHL2870 (IbpA) may be more important in pea bacteroids as *ibpA* expression was about 15-fold up-regulated in RlvA34 pea bacteroids, but only about 2-fold up-regulated in bean bacteroids (Table S4). Numerous export systems that are up-regulated in pea but not bean bacteroids may remove unwanted and/or toxic compounds; these include two pea-specific GSTs (RHL1557 and RHL3865, sharing 32 % id), which were up-regulated about 35- and 10-fold in pea RlvA34 bacteroids (Table S15). Amongst those genes with the most highly elevated expression in pea but not bean bacteroids were the RND family efflux systems encoded by *RHL2076*, *RHL2565–6 RHL2619–20* (*mexF1*), *RHL2967* and *RHL3012* (about 5- to 8-fold up-regulated, Table S12, Fig. 3), a HlyD family efflux pump, *RHL2518* (about 10-fold), contiguous with an ABC export system *RHL2519–21* (about 4- to 6-fold), a TetR family transcriptional regulator (*RHL2517,* about 8-fold), another ABC export cluster *RHL2324* (about 8-fold) and MFS (major facilitator subfamily) protein *RHL2967* (about 5-fold). [For a full list of the 12 genes encoding proteins involved in export (Riley code 1.5.5) that are >5-fold elevated in pea bacteroids and <2-fold elevated in bean bacteroids, see Table S15.] Table S12 and Fig. 4 show up-regulated transporters that are also highly expressed.

##### Central carbon metabolism

In pea bacteroids, strongly up-regulated genes included those encoding TCA (tricarboxylic acid) cycle enzymes RHL1075 (GltA, citrate synthase), RHL3358 (AcnA, aconitase), RHL3260 and RHL3258 (SucAB, oxoglutarate dehydrogenase), RHL3261 (SucD, succinyl-CoA synthetase), RHL3268 (SdhA) and RHL3267 (SdhB) (both parts of the succinate dehydrogenase complex) and RHL3264 (Mdh, malate dehydrogenase) (Table S17, Fig. 5) were also seen in bacteroids of Rlv3841 [13]. In Rlp4292, these genes were up-regulated at most about twofold, except for citrate synthase (GltA) encoded by *RHL1075*, which was up-regulated about fourfold (Table S17), while *RHL1300* (*gltA2*) and *RHL1301* (*citZ*) were unchanged or twofold down-regulated, respectively. From these data, we can predict that bean bacteroids maintain a level of TCA cycle enzymes similar to that of their free-living cells, including glutamine/glutamate synthesis, whereas highly differentiated pea bacteroids have more perturbed central metabolism. It should be stressed that an increase in expression of genes of the TCA cycle does not give information on the flux through the cycle.

The glyoxylate cycle is unlikely to be elevated in pea or bean bacteroids as the genes encoding enzymes RHL4371 (AceA) and RL3631 (GlcB) (Fig. 5) were not differentially expressed in either Rlp4292 or RlvA34 bacteroids (Table S17). Aconitase and 2-ketoglutarate dehydrogenase enzymes are not required for nitrogen fixation in bacteroids of *B. japonicum* [79, 80].

GabD2T, a 2-oxoglutarate dehydrogenase-dependent γ-aminobutyrate (GABA) aminotransferase, converts GABA into glutamate and succinate, which feeds directly into the TCA cycle (Fig. 5). As observed previously [63, 81], expression of *gabDTR* (*RHL3680–2*) was up-regulated in pea bacteroids (about 80-, 65- and 7-fold, respectively), but this was not seen in bean bacteroids (Table S17) suggesting GABA metabolism is important only in pea nodules. GABA from pea nodules can be taken up using the Bra system [58], whose component genes were up-regulated in both pea and bean bacteroids (Table S12, Fig. 4).

#### (iv) Shared genes down-regulated in both bean and pea bacteroids

Table S18 shows 17 genes that are down-regulated >5-fold in both bean and pea bacteroids (summarized in Fig. 3) and these include the ribonucleoside reductase genes *RHL3048–50* (*nrdEIH*) (down-regulated about 5- to-10-fold) that produce dNTPs required for DNA synthesis [82]. The six genes, *RLHa5121–6* (*vbsLDAGSOP*), encoding the iron-scavenging siderophore vicibactin and proteins for its biosynthesis and export, were among the most down-regulated (about 5- to 10-fold) in both bean and pea bacteroids (Table S18). While these genes were down-regulated by approximately the same amount in both bean and pea, the fact that the RlvA34 strain had about fivefold more expression in free-living cells than in those of Rlp4292 (Table S5) means that the absolute expression in RlvA34 bacteroids in pea is about five times higher than in Rlp4292 bean bacteroids. The down-regulation of these genes in bacteroids is indicative of a regulatory mechanism epistatic to RirA, mutation of which caused their increased expression in free-living RlvA34. Other genes thought be affected by the *rirA* mutation in RlvA34 (Table S5) behaved differently; *RHL2531–40* encoding haemin iron transport proteins were slightly down-regulated in bean bacteroids (about 2-fold) but not differentially regulated in RlvA34 pea bacteroids (compared to the elevated level in free-living bacteria); *RHLa5475–7*, encoding a FeCT ABC transporter, was down-regulated in both bean and pea bacteroids by about 3-fold (meaning that the absolute level in RlvA34 pea bacteroids was about 2- to 5-fold higher that in Rlp4292 bean bacteroids) (Table S5). However, it is important to realise that RirA may act differently in bacteroids than in free-living bacteria and this should, therefore, be taken into account.

Three genes strongly down-regulated in both pea and bean bacteroids were the quorum-sensing regulators encodes *cinI* and *cinS,* and the adjacent gene (*RHL2191*) encoding a putative 3-hydroxybutyryl-CoA dehydrogenase (25 % id to HbdA). R*HL2814*, encoding the chemotaxis transcriptional regulator CheY (98 % to RL4036), was down-regulated about fivefold, befitting an environment where chemotaxis is not possible. The polysaccharide lyase, encoded by *RHL1848* (*plyA2*), is down-regulated about 5- to 15-fold in bean and pea bacteroids (Table S18); this lyase cleaves acidic extracellular polysaccharide [83] minimising the high viscosity caused by extracellular polysaccharide (an issue that would be unimportant in bacteroids).

#### (v) Shared genes down-regulated only in bean bacteroids

Table S19 shows 14 genes that were >5-fold down-regulated in bean but not pea bacteroids (summarized in Fig. 3). These include genes encoding hypothetical proteins of unknown function: *RHL0036*, *RHL0040* and *RHL0044,* down-regulated >5-fold in beans, but unchanged in pea bacteroids, and *RHLa4808–9*, *RHLa4834–5* and *RHL5558–60* (Table S19).

#### (vi) Shared genes down-regulated only in pea bacteroids

There are 18 shared genes that are >5-fold down-regulated in pea but not bean bacteroids (Table S20, summarized in Fig. 3). The gene encoding septum site-determining protein MinD (RHLb5723) was down-regulated about sevenfold in Rlv34 pea, but not Rlp4292 bean bacteroids (Table S20), consistent with endoreduplication without cell division in pea bacteroids. Other cell-division-related genes were down-regulated in pea but not bean nodules: *minE* (*RHLb5722*) about 3-fold; *minC* (*RHLb5724*) about 5-fold; *ftsZAQ* (*RHL2104–6*) about 3- to 4-fold; a DNA translocase gene (*RHL3744*) about 3-fold; and *RHL1899* (*ftsZ2*) about 2-fold (Table S4).

Genes encoding FMN reductases, *RHLc6323* and *RHLc6678,* were down-regulated about 5- to 8-fold in RlvA34 pea, but not Rlv4292 bean bacteroids (Table S20). The *RHLb5787–9* genes, encoding a trifolitoxin-related protein and two hypothetical proteins, were down-regulated about fivefold in RlvA34 pea but slightly up-regulated in bean bacteroids.

Genes encoding components of several solute uptake systems were down-regulated in pea but not bean bacteroids; *RHL1566* (NitT), *RHL3478* and *RHLc6677* (PAAT), *RHLb6112* (SulT), *RHLc6283* and *RHLc6625* (CUT2), and *RHLc6332–3* (unclass) (about fivefold). These genes were more highly expressed due to solutes present in the liquid media and the observation that they were not differentially regulated in bean, correlates with the wide-range of solutes present in bean nodules apparent from induction of the large number and variety of bean-specific transporters (Fig. 4).

### Conclusions

(1) Against a general pattern of down-regulation of gene expression in bacteroids compared with free-living rhizobia, both bean and pea bacteroids showed increased expression of genes associated with nitrogen fixation and utilization of dicarboxylates, formate, amino acids and quaternary amines. The decreased expression of many genes may be associated with the increased expression of stringent response genes. (2) Gene expression patterns suggest that bean bacteroids were more limited for P, S and Fe than pea bacteroids, and the expression of cytochrome oxidase genes suggests that bean bacteroids are exposed to higher levels of oxygen than pea bacteroids. (3) Bacteroids in bean nodules expressed many genes whose products are predicted to be related to metabolite detoxification and export. (4) Bean bacteroids express high levels of phasin genes that are associated with storage of the large amounts of PHB that accumulate in bean nodules. (5) Pea nodules have strongly up-regulated genes associated with central metabolism and this is indicative of some fundamental differences in the use of the TCA cycle. (6) Surprisingly, pea bacteroids showed evidence for expression of nitrate and nitrite reduction enzymes. (7) Expression patterns of uptake transporters are indicative that bacteroids in pea and bean nodules are exposed to different sets of substrates specific to each nodule type.

## Data Bibliography

1. The genome sequence of *R. leguminosarum* bv. *phaseoli* 4292 is available from the JGI database; taxon ID 2516653085 (https://img.jgi.doe.gov/cgi-bin/w/main.cgi?section=TaxonDetail&taxon_oid=2516653085).
2. The genome sequence of *R. leguminosarum* bv. *viciae* 248 is available from the JGI database; taxon ID 2515075009 (https://img.jgi.doe.gov/cgi-bin/w/main.cgi?section=TaxonDetail&taxon_oid=2515075009).
3. RNA-Seq data was submitted to the European Bioinformatics Institute (EBI) database (www.ebi.ac.uk/) and details of samples and accession codes (study accession code PRJEB28599) are given in Table S2.
