# Supplementary material for: Transcriptomic analysis of Rhizobium leguminosarum bacteroids in determinate and indeterminate nodules
Source: Microb Genom. 2019 Feb 19;5(2):e000254. doi: 10.1099/mgen.0.000254 (PMC6421345; doi:10.1099/mgen.0.000254)
Supplement: Supplementary File 1 [file mgen-5-254-s001.pdf]

Fig. S1. Replicons of strains Rlp4292 and RlvA34. Shared and unique replicons are drawn in a Venn diagram (not to scale).

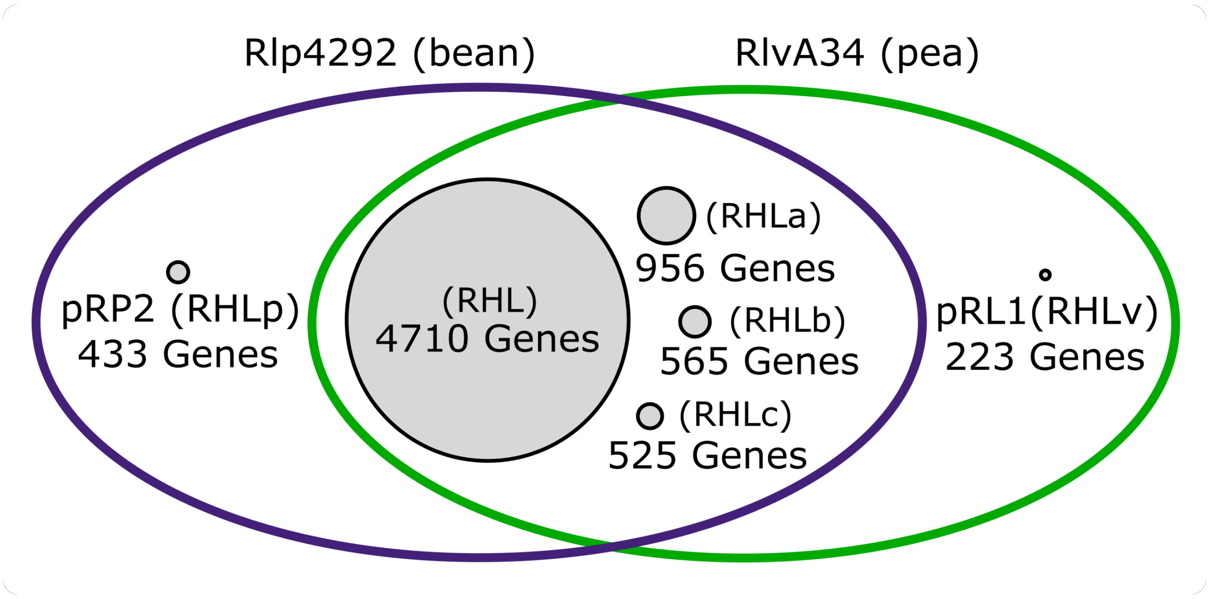

Fig. S2. Genes differentially expressed in bacteroids of Rlp4292 in bean nodules and RlvA34 in pea nodules. Pie charts show, by replicon, the proportion of genes up-regulated >2-fold in green, those not differentially expressed (<2-fold up- or down-regulated) in yellow and down-regulated >2-fold in red. The total number of genes on each replicon is shown. This figure is drawn from data in Table S4.

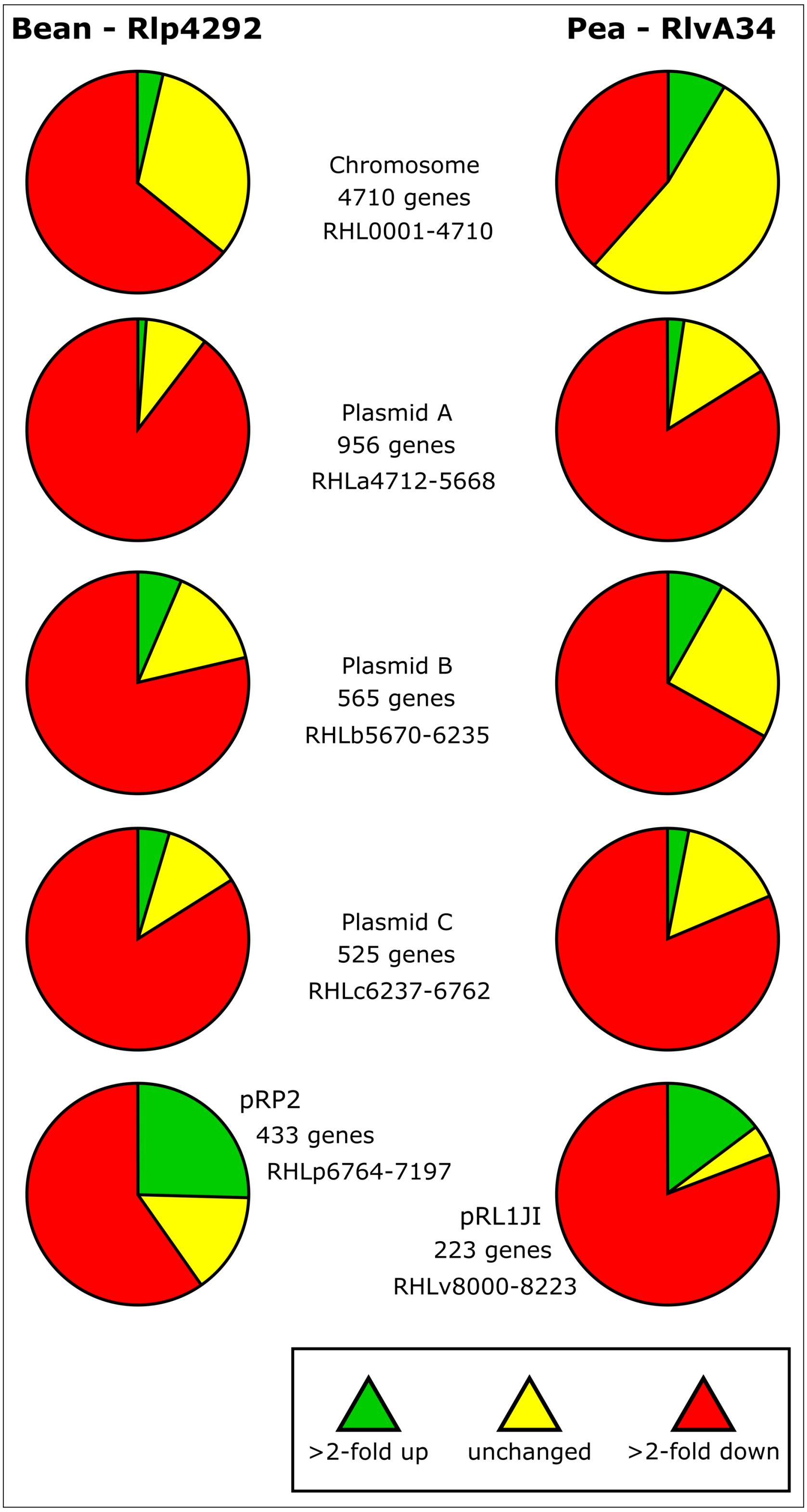

Table S3. Comparison of the multiple copies of Fix, Nif and Nod proteins encoded on the Sym plasmid pRP2 of Rlp4292 with those of Rlv3841.

			% Identity to Rlv3841 gene product:					
Gene product			FixN	NifB	NifD	NifH	NodA	NodD
	Gene		pRL100205	pRL100195	pRL100161	pRL100162	pRL100185	pRL100184
		Amino acids	540	497	501	297	310	540
FixN	RHLp7186	540	93					
ψFixN	RHLp7176	67	29					
NifB1	RHLp7097	492		69				
ψNifB1	RHLp7023	291		60				
ψNifB2	RHLp7057	165		44				
NifD	RHLp7061	500			84			
ψNifD	RHLp7132	70			68			
NifH1	RHLp7062	297				84		
NifH2	RHLp7131	297				89		
ψNifH	RHLp7071	192				93		
NodA1	RHLp7065	195					65	
NodA2	RHLp7104	225					54	
NodD1	RHLp7142	314						69
NodD2	RHLp7160	318						71
NodD3	RHLp7161	302						67

Table S7. Differential gene expression in bacteroids isolated from bean (Rlp4292) and pea (RlvA34) nodules

	Number of genes differentially expressed (>5-fold up- or down-regulated) in bacteroids per replicon				
	Chromosome	Plasmid A	Plasmid B	Plasmid C	Sym plasmid
Genes per replicon	4710	956	565	525	433/223
>5-fold up bean only	183	14	17	22	117
>5-fold up both	56	3	25	5	-
>5-fold up pea only	200	12	13	5	33
>5-fold down bean only	44	40	9	33	12
>5-fold down both	9	7	0	1	-
>5-fold down pea only	18	3	8	15	75
% age of genes per replicon					
>5-fold up bean only	5%	2%	7%	5%	27%
>5-fold up pea only	5%	2%	7%	2%	15%
>5-fold down bean only	1%	5%	2%	6%	3%
>5-fold down pea only	1%	1%	2%	3%	34%
